# High aspartate aminotransferase to alanine aminotransferase ratio on admission as risk factor for poor prognosis in COVID-19 patients

**DOI:** 10.1038/s41598-020-73575-2

**Published:** 2020-10-05

**Authors:** Cheng Qin, Yingxin Wei, Xiaoyu Lyu, Bangbo Zhao, Yunlu Feng, Tianhao Li, Hongtao Cao, Xiaoying Yang, Xingtong Zhou, Weibin Wang, Lei You, Yujun Wang

**Affiliations:** 1 Department of General Surgery, Peking Union Medical College Hospital, Chinese Academy of Medical Sciences, Beijing, 100730 China; 2 Department of Endocrinology, The Central Hospital of Wuhan, Tongji Medical College, Huazhong University of Sciences and Technology, Wuhan, 430014 Hubei Province China; 3 Department of Gastroenterology, Peking Union Medical College Hospital, Chinese Academy of Medical Sciences, Beijing, 100730 China; 4 Department of Surgery, Peking Union Medical College Hospital, Chinese Academy of Medical Sciences, Beijing, 100730 China; 5 Department of Critical Care Medicine, The Central Hospital of Wuhan, Tongji Medical College, Huazhong University of Sciences and Technology, Wuhan, 430014 Hubei Province China

**Keywords:** Biochemistry, Microbiology, Diseases, Gastroenterology, Health care, Risk factors

## Abstract

This study aimed to analyze aspartate aminotransferase (AST) to alanine aminotransferase (ALT) ratio in COVID-19 patients. After exclusion, 567 inpatients were included in this study and separated into two groups according to their AST/ALT ratio on admission. Death was regarded as poor prognosis in this study. Of 567 patients, 200 (35.3%) had AST/ALT ≥ 1.38. Of the 200 patients, older age (median age 60 years), myalgia (64 [32%] cases), fatigue (91 [45.5%] cases), some comorbidities and outcomes were significantly different from patients with AST/ALT < 1.38. They also had worse chest computed tomography (CT) findings, laboratory results and severity scores. Levels of platelet count (OR 0.995, 95% CI [0.992–0.998]) and hemoglobin (OR 0.984, 95% CI [0.972–0.995]) were independently associated with AST/ALT ≥ 1.38 on admission. Furthermore, a high AST/ALT ratio on admission was an independent risk factor for poor prognosis (OR 99.9, 95% CI [2.1–4280.5]). In subsequent monitoring, both survivors and non-survivors showed decreased AST/ALT ratio during hospitalization. In conclusion, high AST/ALT ratio might be the indication of worse status and outcomes in COVID-19 patients.

## Introduction

Coronaviruses, consisting of four genera (*Alphacoronavirus*, *Betacoronavirus*, *Gammacoronavirus* and *Deltacoronavirus*), are members of the *Coronaviridae* family. In contrast to the majority of human coronaviruses that only cause mild respiratory infections, highly pathogenic coronaviruses can induce severe human respiratory syndromes and threaten public health^1^. In 2002 and 2003, the worldwide outbreak of severe acute respiratory syndrome (SARS), which is caused by SARS coronavirus (SARS-CoV), infected over 8400 people in at least 30 countries and resulted in more than 800 reported deaths (approximately 10% mortality)^2^. Ten years later, Middle East respiratory syndrome coronavirus (MERS-CoV) spread in 27 countries and caused 779 deaths in 2182 MERS cases (approximately 35% mortality)^3^. At present, the world is experiencing a pandemic of coronavirus disease 2019 (COVID-19), which is caused by SARS-CoV-2 and has contributed to far more cases and deaths than SARS and MERS^4^.

SARS-CoV-2 is a novel RNA virus and belongs to the *Betacoronavirus* genus. Its genome sequence shares approximately 79% identity with SARS-CoV and 50% identity with MERS-CoV^5^. Notably, SARS-CoV-2 employs angiotensin-converting enzyme 2 (ACE2) as a host-cell entry receptor, which is similarly used by SARS-CoV^6^. In December 2019, the first series of cases were confirmed in Wuhan, Hubei Province, China^7^. Since then, COVID-19 has affected over 200 countries and infected over twenty million people, with over eighty hundred thousand deaths as of August 2020. According to the clinical investigation of COVID-19 patients, fever and respiratory symptoms predominate^8–10^. In addition, a recent study found that digestive symptoms, including abdominal pain, diarrhea, vomiting and nausea, are also present in 26% of COVID-19 patients^11^. In addition to clinical evidence, single-cell analysis data suggested that some cells in the digestive system also express ACE2, which might mediate SARS-CoV-2 infection in the liver^12^ and gut^13,14^. Furthermore, a retrospective study involving 191 patients showed that sepsis was the most frequent complication^15^, which could cause systemic damage, including liver injury^16^. Therefore, liver might play a significant role in the course of COVID-19. Clinical studies have reported that approximately 20%-30% of COVID-19 patients have liver dysfunction, which is represented by elevated levels of alanine aminotransferase (ALT) or aspartate aminotransferase (AST)^8,17,18^. Both ALT and AST are concentrated in liver, and elevations in their levels are two routinely clinical indexes indicating hepatic damage. Compared to the diffused expression of AST in other tissues as well, ALT is more specifically originated from liver. Therefore, increased ALT levels in serum have been considered more specific for liver damage than AST^19^. Whereas in liver, AST is especially located in the zone 3 of acinus and the hepatocellular mitochondrial^19^. Therefore, damage to zone 3, or worse hepatocyte injuries involving mitochondrial, may result in greater elevation to AST levels^19,20^. In addition to individual values of ALT and AST, the AST/ALT ratio has been explored as an important indicator for assessing liver and other diseases. An elevated AST/ALT ratio might indicate alcoholic liver disease, cirrhosis^21^, and poor prognosis in acute viral hepatitis^22^. Although higher AST levels are likely to associate with death in COVID-19 patients^23^, there is still a lack of clinical studies on AST/ALT ratio to predict disease course in COVID-19 patients.

In this study, we described the clinical characteristics, laboratory results and outcomes of COVID-19 patients with AST/ALT < 1.38 and ≥ 1.38 on admission in Wuhan, Hubei Province. Furthermore, the changes in AST/ALT ratio during hospitalization were analyzed. In conclusion, higher AST/ALT ratio indicates worse clinical status and poor prognosis and should be considered by clinicians during the treatment of COVID-19 patients.

## Results

### AST/ALT ratio could predict the prognosis of COVID-19 patients

**Figure 1:**
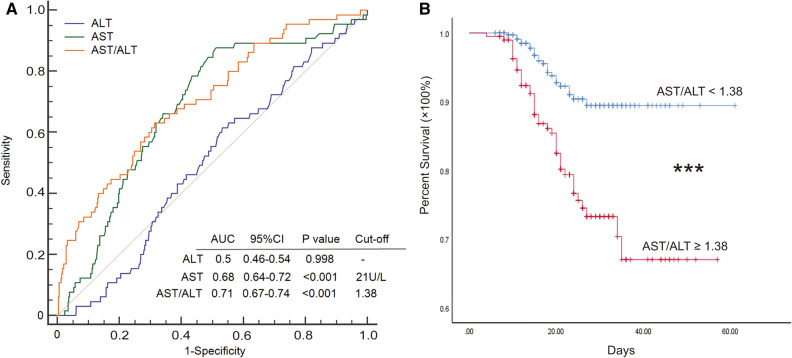
AST/ALT ratio distinguished COVID-19 patients with different prognosis. Only patients with clear endpoints (recovery and death) were involved in the analysis about prognosis (n = 555). (**A**) Receiver operating curve (ROC) was utilized to compare the ability of ALT, AST, and AST/ALT ratio to predict death in hospitalized COVID-19 patients. Cut-off values were determined through Youden index. (**B**) Kaplan–Meier survival analysis in patients with AST/ALT < 1.38 and ≥ 1.38. *AUC* area under the curve; ***P < 0.001.

In overall 567 patients, after excluding 12 patients who were transferred to other hospitals with unknown outcomes, 555 patients with clear endpoints (recovery or death) were involved in the analysis about prognosis. To determine the ability of different clinical indexes in predicting prognosis, we drew receiver operating curves (ROC) of ALT, AST, and AST/ALT to predict death. Among them, both AST levels (P < 0.001) and AST/ALT ratio (P < 0.001) could effectively distinguish different prognosis in hospitalized COVID-19 patients. After balancing sensitivity and specificity, the cut-off value of AST/ALT ratio was determined as 1.38 (Fig. 1A). Moreover, patients with AST/ALT ≥ 1.38 on admission had significantly poor survival when compared to those with AST/ALT < 1.38 (P < 0.001) (Fig. 1B).

### Clinical features

567 hospitalized patients with confirmed COVID-19 were included in analyzing clinical features. The median age was 55 years (IQR, 37–67), and 247 (43.6%) patients were male. The median duration from symptom onset to hospital admission was 7 days (IQR, 4–10). The most common symptoms on admission were fever (455 [80.2%]), cough (370 [65.3%]), chest tightness (222 [39.2%]), fatigue (208 [36.7%]), and myalgia (143 [25.2%]). In contrast, the less common symptoms of the included patients were diarrhea (51[9%]), headache (36 [6.3%]), and chest pain (27 [4.8%]). Of the 567 patients, 250 (44.1%) had coexisting diseases, 108 (19%) had ≥ 2 comorbidities. A total of 183 (32.3%) patients had hypertension, 85 (15%) patients had diabetes, and 53 (9.3%) patients had cardiovascular disease, which were the most common comorbidities. In contrast, chronic obstructive pulmonary disease (36 [6.3%]), cerebrovascular disease (32 [5.6%]), and chronic kidney disease (31 [5.5%]) were less common comorbidities (Table 1).

**Table 1:** Clinical characteristics of the included COVID-19 patients.

Characteristics	All patients (n = 567)	AST/ALT < 1.38 (n = 367)	AST/ALT ≥ 1.38 (n = 200)	P Value
Age, median (IQR), years	55 (37–67)	51 (37–64)	60 (37.3–71)	0.001*
**Sex**				0.278
Male	247 (43.6)	166 (45.2)	81 (40.5)	
Female	320 (56.4)	201 (54.8)	119 (59.5)	
**Time**				
Illness onset to hospital admission, median (IQR), days	7 (4–10)	7 (4–10)	7 (3–9)	0.059
**Signs and symptoms**				
Fever	455 (80.2)	293 (79.8)	162 (81)	0.74
Myalgia	143 (25.2)	79 (21.5)	64 (32)	0.006*
Fatigue	208 (36.7)	117 (31.9)	91 (45.5)	0.001*
Headache	36 (6.3)	22 (6)	14 (7)	0.639
Cough	370 (65.3)	238 (64.9)	132 (66)	0.784
Chest tightness	222 (39.2)	144 (39.2)	78 (39)	0.956
Chest pain	27 (4.8)	19 (5.2)	8 (4)	0.529
Diarrhea	51 (9)	33 (9)	18 (9)	0.997
**Comorbidities**				
Any	250 (44.1)	154 (42)	96 (48)	0.166
COPD	36 (6.3)	17 (3)	19 (9.5)	0.023*
Hypertension	183 (32.3)	115 (31.3)	68 (34)	0.517
Diabetes	85 (15)	50 (13.6)	35 (17.5)	0.217
Cardiovascular disease	53 (9.3)	25 (6.8)	28 (14)	0.005*
Cerebrovascular disease	32 (5.6)	16 (4.4)	16 (8)	0.073
Chronic kidney disease	31 (5.5)	12 (3.3)	19 (9.5)	0.002*
≥ 2 comorbidities co-exist	108 (19)	58 (15.8)	50 (25)	0.008*
**Outcomes**				
Recovery and discharge	490 (86.4)	336 (91.6)	154 (77)	< 0.001*
Death	65 (11.5)	24 (6.5)	41 (20.5)	< 0.001*
Transfer to specialized hospitals	12 (2.1)	7 (1.9)	5 (2.5)	0.87

Values are numbers (percentages) unless stated otherwise.

*A two-tailed P value less than 0.05 was considered statistically significant.

Of the 567 included patients, 200 patients (35.3%) had AST/ALT ≥ 1.38. Compared with the patients with AST/ALT < 1.38 on admission (n = 367, 64.7%), patients with AST/ALT ≥ 1.38 were older (median age, 60 years [IQR, 37.3–71] vs 51 years [IQR, 37–64]; P = 0.001), more likely to have myalgia (64 [32%] vs 79 [21.5%]; P = 0.006), fatigue (91 [45.5%] vs 117 [31.9%]; P = 0.001) and some coexisting diseases, such as COPD (19 [9.5%] vs 17 [3%]; P = 0.023), cardiovascular diseases (28 [14%] vs 25 [6.8%]; P = 0.005) and chronic kidney diseases (19 [9.5%] vs 12 [3.3%]; P = 0.002), on admission. Interestingly, patients with AST/ALT ≥ 1.38 preferred to have ≥ 2 comorbidities on admission (50 [25%] vs 58[25%]; P = 0.008). Additionally, patients with AST/ALT ≥ 1.38 on admission were less likely to be recovery and discharge (154 [77%] vs 336 [91.6%]; P < 0.001) but more likely to be death (41 [20.5%] vs 24 [6.5%]; P < 0.001) (Table 1).

### AST/ALT ≥ 1.38 on admission indicates more severe chest CT findings, worse laboratory results and higher severity of illness scores

On admission, all enrolled patients experienced a variety of measures and tests according to their clinical care needs. There were differences on chest CT, in laboratory findings and in severity of illness scores between patients with AST/ALT < 1.38 or ≥ 1.38 (Table 2).

**Table 2:** Chest CT findings, laboratory results and severity of illness scores of COVID-19 patients on admission.

	All patients (n = 567)	AST/ALT < 1.38 (n = 367)	AST/ALT ≥ 1.38 (n = 200)	P Value
**Chest CT, numbers (percentages)**				
Lesions occupy < 30% lung	309/554 (55.8)	211/360 (58.6)	98/194 (50.5)	0.067
Lesions occupy 30%-60% lung	115/554 (20.8)	75/360 (20.8)	40/194 (20.6)	0.953
Lesions occupy > 60% lung	130/554 (23.5)	74/360 (20.5)	56/194 (28.9)	0.028*
**Laboratory findings**				
White blood cell count, × 109/L	4.9 (3.7–6.5)	5 (3.9–6.5)	4.6 (3.4–6.4)	0.024*
Neutrophil count, × 109/L	3.2 (2.2–4.7)	3.4 (2.3–4.8)	3.1 (2–4.6)	0.123
Lymphocyte count, × 109/L	1 (0.7–1.4)	1.1 (0.7–1.4)	0.9 (0.6–1.2)	0.004*
Hemoglobin, g/L	128 (119.8–140)	129 (121–141)	127 (114–138)	0.001*
Platelet count, × 109/L	178 (134–225)	186 (146–239)	159 (118–201)	< 0.001*
Total bilirubin, μmol/L	8.5 (6.4–11.9)	8.8 (6.6–11.9)	8.1 (5.5–11.7)	0.068
Blood urea nitrogen, mmol/L	4.1 (3.2–5.4)	4 (3.3–5.2)	4.2 (3.2–5.9)	0.302
Creatinine, μmol/L	64.4 (51.8–78.1)	63.8 (50.8–77.2)	65.5 (53.6–79.6)	0.088
Lactate dehydrogenase, U/L^¶^	188 (150.3–251.8)	182 (148–236.3)	203 (158.8–290.3)	0.002*
Creatinine kinase, U/L^¶^	77 (48–139)	74.7 (49–126)	85.6 (46.8–169)	0.128
Creatine kinase–MB, U/L^§^	8 (6–12)	7 (6–11)	9 (6–12.9)	0.008*
C-reactive protein, mg/dL	1.6 (0.4–4.4)	1.4 (0.3–3.7)	2.5 (0.5–6.4)	0.002*
Procalcitonin, ng/mL^†^	0.05 (0.04–0.09)	0.05 (0.04–0.08)	0.06 (0.04–0.135)	0.004*
IL-6 pg/mL^‡^	4.7 (2.3–17.9)	4 (2.3–13.3)	7 (2.3–27.3)	0.246
D-dimer, μg/mL	0.5 (0.3–1.1)	0.5 (0.2–1)	0.6 (0.3–1.2)	0.111
Fibronectin g/L	3 (2.5–3.5)	2.9 (2.5–3.5)	3 (2.6–3.5)	0.068
Lactate, mmol/L	1.2 (0.8–1.9)	1.2 (0.8–1.8)	1.2 (0.8–1.9)	0.697
PaO_2_/FiO_2_, mm Hg	363 (243–520)	400 (270–520)	326.5 (202–480.5)	< 0.001*
**Severity of illness scores**				
APACHE II	3 (1–5)	2 (1–4)	4 (2–8)	< 0.001*
SOFA	1 (0–3)	1 (0–2)	2 (0–3)	< 0.001*
CURB65, mean ± SD	0.56 ± 0.81	0.45 ± 0.7	0.76 ± 0.94	< 0.001*

Values are medians (interquartile ranges) unless stated otherwise.

*A two-tailed P value less than 0.05 was considered statistically significant.

Missing data for over 5% of patients are indicated:

^¶^Data on LDH and creatine kinase were missing for 47 patients (8.3%), including 33 patients with AST/ALT < 1.38 (9%) and 14 patients with AST/ALT ≥ 1.38 (7%).

^§^Data on creatine kinase-MB were missing for 42 patients (7.4%), including 31 patients with AST/ALT < 1.38 (8.4%) and 11 patients with AST/ALT ≥ 1.38 (5.5%).

^†^Data on procalcitonin were missing for 45 patients (7.9%), including 30 patients with AST/ALT < 1.38 (8.2%) and 15 patients with AST/ALT ≥ 1.38 (7.5%).

^‡^Data on creatine IL-6 were missing for 413 patients (72.8%), including 268 patients with AST/ALT < 1.38 (73%) and 145 patients with AST/ALT ≥ 1.38 (72.5%).

Compared with the patients with AST/ALT < 2, patients with AST/ALT ≥ 2 were more likely to have lesion presence higher than 60% lung (56 [28.9%] vs 74 [20.5%]; P = 0.028) (Table 2). Among laboratory findings, the blood count showed that patients AST/ALT ≥ 2 had lower white blood cell count (median number, 4.6 × 10^9^/L [IQR, 3.4–6.4] vs 5 × 10^9^/L [IQR, 3.9–6.5]; P = 0.024), lymphocyte count (median number, 0.9 × 10^9^/L [IQR, 0.6–1.2] vs 1.1 × 10^9^/L [IQR, 0.7–1.4]; P = 0.004), hemoglobin (median number, 127 g/L [IQR, 114–138] vs 129 g/L [IQR, 121–141]; P = 0.001) and platelet count (median number, 159 × 10^9^/L [IQR, 118–201] vs 186 × 10^9^/L [IQR, 146–239]; P < 0.001) than those with AST/ALT < 1.38. In the myocardial enzyme spectrum test, patients with AST/ALT ≥ 1.38 had slightly higher CK-MB (median number, 9 U/L [IQR, 6–12.9] vs 7 U/L [IQR, 6–11]; P = 0.008), and LDH (median number, 203 U/L [IQR, 158.8–290.3] vs 182 U/L [IQR, 148–236.3]; P = 0.002). The abovementioned differences suggest that patients with AST/ALT ≥ 1.38 are more likely to encounter systemic injury, including the circulatory system and heart. Consistently, some parameters of systemic inflammation were also significantly increased in the patients with AST/ALT ≥ 1.38, including CRP (median number, 2.5 mg/dL [IQR, 0.5–6.4] vs 1.4 mg/dL [IQR, 0.3–3.7]; P = 0.002) and procalcitonin (median number, 0.06 ng/mL [IQR, 0.04–0.135] vs 0.05 ng/mL [IQR, 0.04–0.08]; P = 0.004).

In addition to massive differences in laboratory findings, some life-threatening signs were also clearly distinct (Table 2). Blood gas analysis showed that compared with patients who had AST/ALT < 1.38, patients with AST/ALT ≥ 1.38 had lower partial pressure of arterial oxygen to fraction of inspired oxygen ratios (PaO_2_/FiO_2_) (median number, 326.5 mmHg [IQR, 202–480.5] vs 400 mmHg [IQR, 270–520]; P < 0.001), which indicates respiratory dysfunction^24^. Furthermore, patients with AST/ALT ≥ 1.38 also had higher severity of illness scores, including APACHE II (median score, 4 [IQR, 2–8] vs 2 [IQR, 1–4]; P < 0.001), SOFA (median score, 2 [IQR, 0–3] vs 1 [IQR, 0–2]; P < 0.001) and CURE-65 (mean ± SD, 0.76 ± 0.94 vs 0.45 ± 0.7; P < 0.001) (Table 2).

### Platelet count and hemoglobin levels were independently associated with AST/ALT ≥ 1.38

To assess the correlations among other laboratory indicators for AST/ALT ≥ 1.38 in COVID-19 patients, we performed logistic regression analysis for significant differences of the abovementioned laboratory parameters, including white blood cell count, lymphocyte count, hemoglobin, platelet count, LDH, CK-MB, CRP, procalcitonin and PaO_2_/FiO_2_ (Table 3). In collinearity diagnostics, all included laboratory parameters had no significant collinearity. In multivariate logistic regression, levels of hemoglobin (adjusted OR 0.984; 95% CI [0.972–0.995]; P = 0.007) and platelet count (adjusted OR 0.995; 95% CI [0.992–0.998]; P = 0.001) were independently associated with AST/ALT ≥ 1.38 in COVID-19 patients on admission (Table 3).

**Table 3:** Multivariate analysis of AST/ALT ≥ 1.38.

Laboratory parameters	Multivariable OR (95% CI)	P Value
White blood cell count	0.94 (0.865–1.023)	0.15
Lymphocyte count	1.055 (0.732–1.519)	0.775
Hemoglobin	0.984 (0.972–0.995)	0.007*
Platelet count	0.995 (0.992–0.998)	0.001*
Lactate dehydrogenase	1.002 (1–1.004)	0.123
Creatine kinase–MB	1.002 (0.982–1.021)	0.877
C-reactive protein	1.044 (0.975–1.119)	0.219
Procalcitonin	1.051 (0.488–2.267)	0.899
PaO2/FiO2	0.999 (0.998–1.001)	0.261

*A two-tailed P value less than 0.05 was considered statistically significant.

### Elevated AST/ALT ratio on admission indicates poor prognosis as an independent risk factor

Of the 567 included patients, 490 (86.4%) of them recovered and were discharged after comprehensive clinical assessment of symptoms, chest CT and viral clearance; 65 (11.5%) patients died during hospitalization; 12 (2.1%) patients with unknown outcomes were transfer to other specialized hospitals due to deterioration. To analyze the risk factors of biochemical findings on poor prognosis, we performed logistic regression analysis in 555 patients with clear survival information (recovery or death) (Table 4). In univariate analysis, in addition to AST/ALT (crude OR 3.62; 95% CI [2.35–5.57]; P < 0.001), high levels of many other serum biochemical parameters were risk factors for poor prognosis as well, including total bilirubin (crude OR 1.06; 95% CI [1.02–1.11]; P = 0.004), BUN (crude OR 1.14; 95% CI [1.09–1.19]; P < 0.001), creatinine (crude OR 1.002; 95% CI [1.001–1.003]; P < 0.001), LDH (crude OR 1.009; 95% CI [1.006–1.011]; P < 0.001), CK-MB (crude OR 1.05; 95% CI [1.01–1.09]; P = 0.006), white blood cell count (crude OR 1.17; 95% CI [1.09–1.26]; P < 0.001), neutrophil count (crude OR 1.24; 95% CI [1.15–1.34]; P < 0.001), lactate (crude OR 2.3; 95% CI [1.75–3]; P < 0.001), D-dimer (crude OR 1.02; 95% CI [1–1.04]; P = 0.018), fibrinogen (crude OR 1.52; 95% CI [1.14–2.03]; P = 0.004), IL-6 (crude OR 1.04; 95% CI [1.02–1.06]; P < 0.001), C-reactive protein (crude OR 1.26; 95% CI [1.19–1.34]; P < 0.001) and procalcitonin (crude OR 69.6; 95% CI [17.6–275.8]; P < 0.001). In contrast, in some parameters, high levels were protective factors, including lymphocyte count (crude OR 0.17; 95% CI [0.08–0.34]; P < 0.001), hemoglobin (crude OR 0.98; 95% CI [0.97–0.99]; P = 0.002) and platelet count (crude OR 0.99; 95% CI [0.98–0.99]; P < 0.001) (Table 4).

**Table 4:** Univariate and multivariate analysis on poor prognosis (Death).

Biochemical parameters	Univariate analysis		Selection process of variables			Multivariate analysis	
	OR	P Value	VIF	Correlat coefficiention	P Value	OR	P Value
**Liver**							
AST/ALT	3.62 (2.35–5.57)	< 0.001*	1.5	–	–	99.9 (2.1–4280.5)	0.02*
Total bilirubin	1.06 (1.02–1.11)	0.004*	1.2	–	–	0.99 (0.88–1.12)	0.916
**Kidney**							
Blood urea nitrogen	1.14 (1.09–1.19)	< 0.001*	7.8	0.34	< 0.001*	1.64 (1.01–2.66)	0.047*
Creatinine	1.002 (1.001–1.003)	< 0.001*	8.2	0.25	< 0.001*	–	–
**Heart**							
Lactate dehydrogenase	1.009 (1.006–1.011)	< 0.001*	2.3	–	–	1.02 (1–1.03)	0.05
Creatinine kinase	1.001 (1–1.002)	0.103	–	–	–	–	–
Creatine kinase–MB	1.05 (1.01–1.09)	0.006*	1.3	–	–	0.79 (0.52–1.19)	0.252
**Blood**							
White blood cell count	1.17 (1.09–1.26)	< 0.001*	18.7	0.11	0.01*		
Neutrophil count	1.24 (1.15–1.34)	< 0.001*	18.2	0.18	< 0.001*	0.66 (0.28–1.54)	0.333
Lymphocyte count	0.17 (0.08–0.34)	< 0.001*	2.7	–	–	0.11 (0.04–2.78)	0.181
Hemoglobin	0.98 (0.97–0.99)	0.002*	1.2	–	–	0.94 (0.87–1.01)	0.103
Platelet count	0.99 (0.98–0.99)	< 0.001*	1.6	–	–	1.01 (0.98–1.04)	0.47
**Microcirculation dysfunction**							
Lactate	2.3 (1.75–3)	< 0.001*	1.3	–	–	30.53 (2.1–444.4)	0.012*
D-dimer	1.02 (1–1.04)	0.018*	1.4	–	–	0.99 (0.95–1.03)	0.493
Fibrinogen	1.52 (1.14–2.03)	0.004*	1.7	–	–	1.46 (0.38–5.66)	0.581
**Systematic inflammation**							
IL-6	1.04 (1.02–1.06)	< 0.001*	1.5	–	–	1.05 (1–1.1)	0.054
C-reactive protein	1.26 (1.19–1.34)	< 0.001*	2.7	–	–	0.92 (0.6–1.4)	0.671
Procalcitonin	69.6 (17.6–275.8)	< 0.001*	2.3	–	–	0.33 (0.004–25.44)	0.618

Patients with clear survival data (recovery and death) were enrolled in the analysis on prognosis (N = 555).

^#^Variance inflation factor (VIF) > 5 was considered collinearity.

*A two-tailed P value less than 0.05 was considered statistically significant.

To assess independent risk factors for poor prognosis, we performed logistic regression analysis on liver enzymes and other biochemical parameters. However, collinearity was significant among BUN, creatinine, white blood cell count and neutrophil count (VIF = 7.8, 8.2, 18.7 and 18.2, respectively). Therefore, we performed Spearman’s rank correlation analysis in order to select variables and reduce collinearity. BUN and neutrophil count had higher correlation coefficients than creatinine (0.34 vs 0.25) and white blood cell count (0.18 vs 0.11), respectively. Therefore, creatinine and white blood cell count were excluded from the subsequent multivariate logistical regression. In multivariate analysis, high AST/ALT ratio (adjusted OR 99.9; 95% CI [2.1–4280.5]; P = 0.02), BUN (adjusted OR 1.64; 95% CI [1.01–2.66]; P = 0.047) and lactate levels (adjusted OR 30.53; 95% CI [2.1–444.4]; P = 0.012) on admission were relatively independent risk factors for poor prognosis of the COVID-19 patients (Table 4).

To assess the AST/ALT ratio on poor prognosis of patients with different liver enzyme levels^23^, we separated the 555 COVID-19 patients with clear survival data (recovery or death) into two groups according to their AST levels (≤ 40 or > 40 U/L) on admission (Table 5). Of the patients with normal liver enzyme levels and AST/ALT < 1.38 (n = 295), 17 (5.8%) patients had poor prognosis, while of the patients with AST/ALT ≥ 1.38 (n = 157), 28 (17.8%) patients had poor prognosis (OR 3.5; 95% CI [1.9–6.7]; P < 0.001). Of the patients with AST levels > 40 U/L and AST/ALT < 1.38 (n = 65), 7 (10.8%) patients had poor prognosis, while of the patients with AST/ALT ≥ 1.38 (n = 38), 13 (34.2%) patients had poor prognosis (OR 4.3; 95% CI [1.5–12]; P = 0.006) (Table 5). Therefore, AST/ALT ≥ 1.38 was a risk factor for poor prognosis in both groups of patients with different AST levels (≤ 40 or > 40 U/L).

**Table 5:** Univariate analysis of the AST/ALT ratio on poor prognosis in the two groups of patients.

Outcomes	Patients with normal AST levels (n = 452)				Patients with AST > 40 U/L (n = 103)			
	AST/ALT < 1.38 (n = 295)	AST/ALT ≥ 1.38 (n = 157)	P Value	OR (95% CI)	AST/ALT < 1.38 (n = 65)	AST/ALT ≥ 1.38 (n = 38)	P Value	OR (95% CI)
Death	17(5.8)	28(17.8)	< 0.001 *	3.5 (1.9–6.7)	7(10.8)	13(34.2)	0.006 *	4.3 (1.5–12)
Recovery and Discharge	278(94.2)	129(82.2)			58(89.2)	25(65.8)		

Values are numbers (percentages) unless stated otherwise.

Patients with clear survival data (recovery and death) were enrolled in the analysis on prognosis (N = 555).

*A two-tailed P value less than 0.05 was considered statistically significant.

### Monitoring AST/ALT ratio during hospitalization

**Figure 2:**
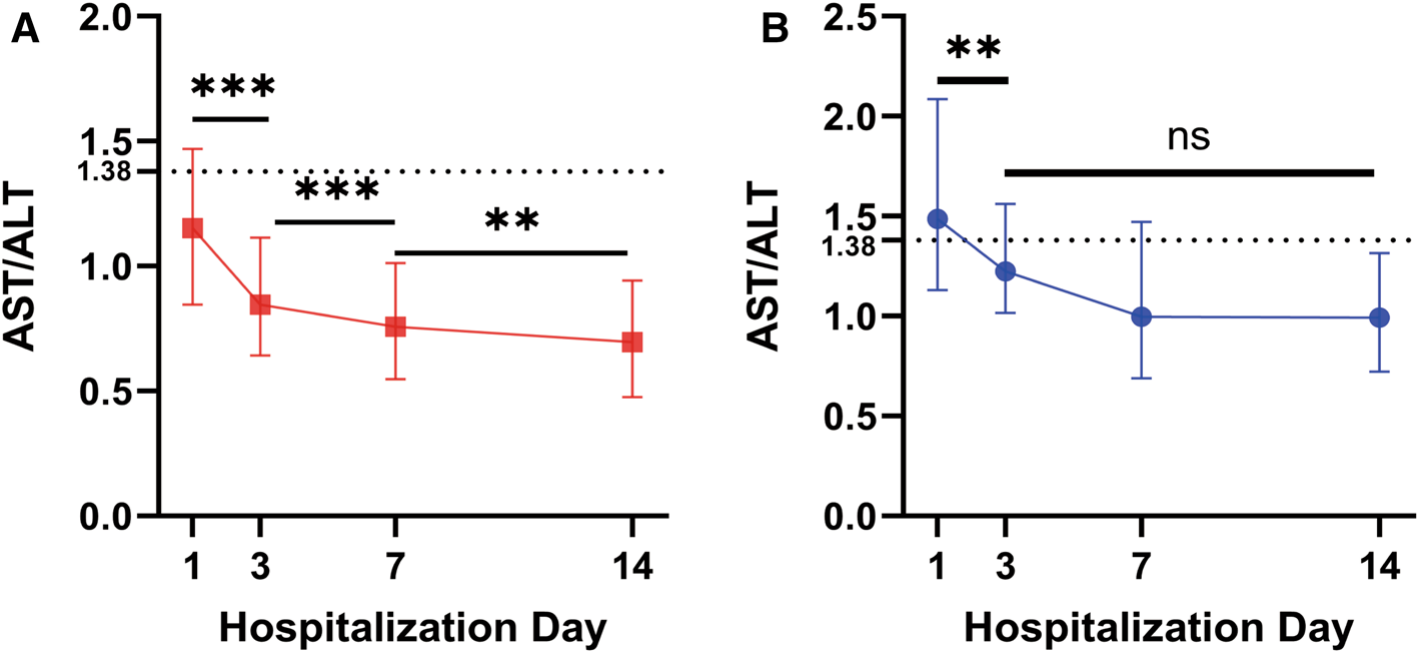
The dynamic changes in AST/ALT ratio in COVID-19 patients during hospitalization. Data were shown as the median (IQR). Paired Wilcoxon's tests were used to compare two neighboring AST/ALT ratio. (A) The AST/ALT ratio of discharged patients who recovered from COVID-19. Data were available for 490 patients (day 1), 225 patients (day 3), 336 patients (day 7) and 279 patients (day 14). (B) The AST/ALT ratio of patients with poor prognosis (death), and data were available for 65 patients (day 1), 28 patients (day 3), 28 patients (day 7) and 17 patients (day 14). ns: no significance; **P < 0.01; ***P < 0.001.

Most patients also received liver enzyme tests on other hospital days (days 3, 7 and 14) in addition to on the day of admission according to their clinical care needs. In discharged patients with recovery, their AST/ALT ratio significantly decreased during hospitalization (Fig. 2A). Similarly, the AST/ALT ratio of patients with poor prognosis also decreased noticeably from day 1 to day 3. From day 3 to day 14, the AST/ALT ratio also tended to decrease, but there was no statistical significance (Fig. 2B).

## Discussion

Among a variety of recent publications about COVID-19, accumulated evidence suggests that SARS-CoV-2 infections might cause multiple and systemic injuries, including acute renal failure, myocardial dysfunction, and acute liver injury^23,25,26^. Additionally, there are recent studies analyzing COVID-19 patients in detail with respect to liver enzymes, such as ALT and AST^23,27^. The underlying mechanism of elevated liver enzymes in COVID-19 patients remains unclear. Additionally, the relationship between COVID-19 and AST/ALT ratio was still not revealed.

In this study, we described COVID-19 patients admitted with different AST/ALT ratio, we found that a high AST/ALT ratio was a risk factor for poor prognosis and monitored changes in AST/ALT ratio during hospitalization. Older COVID-19 patients had a higher case fatality rate^28^. Similarly, having comorbidities might also contribute to worse outcomes^29^. In this study, there was a higher proportion of older and comorbid patients with AST/ALT ≥ 1.38. COPD, cardiovascular, chronic kidney diseases were the most significant coexisting diseases among the 6 comorbidities of patients with AST/ALT ≥ 1.38 on admission. Interestingly, they also preferred to have more than one comorbidity, which might be related with their worse status. Additionally, patients with AST/ALT ≥ 1.38 on admission were also likely to have poor prognosis (death). Therefore, liver tests on elderly COVID-19 patients with myalgia, fatigue and ≥ 2 comorbidities are necessary on admission.

The relationship between AST/ALT ratio and other laboratory findings in COVID-19 patients is unclear. This study described massive differences in laboratory parameters between the two groups of patients. Patients with AST/ALT ≥ 1.38 had lower hemoglobin, lymphocyte and platelet counts, which might be related to the disease severity^30,31^. As previous reports, increased white blood cell counts also indicated patients with severe COVID-19^30,32^. However, our results suggested that patients with AST/ALT ≥ 1.38 were likely to have lower white blood cell counts, which seems to be against previous reports. Interestingly, when compared the white blood cell counts between survivors and non-survivors in our study, patients with poor prognosis indeed had higher white blood cell counts (median number, 6.02 × 10^9^/L [IQR, 3.7–9.4] vs 4.8 × 10^9^/L [IQR, 3.7–6.2]; P = 0.01). Therefore, the relationship between AST/ALT ratio and white blood cell counts remains further research.

Furthermore, compared with patients with AST/ALT < 1.38, those patients with AST/ALT ≥ 1.38 were also susceptible to have worse myocardial function (higher LDH and CK-MB levels) and more severe systemic inflammation (higher CRP and procalcitonin levels). In general, AST/ALT ≥ 1.38 might indicate that the patients were suffering from more intensive systemic injuries. In addition, more severe chest CT results (showing a larger proportion of lesions in the lung), worse blood gas analysis (lower PaO_2_/FiO_2_) and higher severity of illness scores (higher APACHE II, SOFA and CURB-65) were also more frequent in patients with AST/ALT ≥ 1.38. Importantly, a higher SOFA score have been recommended as indictors for worse outcomes in early COVID-19 patients^15^. Moreover, in multivariate analysis, lower levels of hemoglobin and platelet count were independently associated with AST/ALT ≥ 1.38.

Additionally, the AST/ALT ratio was found to be a risk factor for death in both groups of COVID-19 patients with normal or abnormal AST levels. Besides that, the AST/ALT ratio, for unclear reasons, has some extrahepatic implications, including predicting poor outcomes of pancreatic cancer patients^33^ and heart injury in Kawasaki disease^34^. Therefore, the role of the AST/ALT ratio in COVID-19 remains to be further analyzed. We also monitored the dynamic AST/ALT ratio during hospitalization. The AST/ALT ratio tended to decrease during hospitalization in both two groups patients with distinct outcomes. Therefore, high AST/ALT ratio in COVID-19 patients might be eased to a large extent after receiving medical treatment. However, the AST/ALT ratio in patients with recovery showed more sustained reduction. Therefore, after day 3, continuously decreased AST/ALT ratio might indicate good prognosis in COVID-19 patients. Future studies on the dynamic changes of AST/ALT ratio in COVID-19 are expected.

In summary, our results suggested that older COVID-19 patients with myalgia, fatigue, or ≥ 2 coexisting diseases were more likely to have AST/ALT ≥ 1.38 on admission. We also provided evidence that AST/ALT ≥ 1.38 was significantly associated with more severe chest CT findings, worse laboratory results, higher severity of illness scores, and poor prognosis as an independent risk factor of COVID-19 patients. Therefore, it is necessary to provide advanced medical care to COVID-19 patients with AST/ALT ≥ 1.38 on admission.

## Methods

### Study design and patients

This retrospective study was approved by the Ethics Committee of The Central Hospital of Wuhan. All methods were conducted in accordance with the Declaration of Helsinki. Written informed consent was obtained from all participants for extracting data from their clinical records. All authors had access to the study data and reviewed and approved the final manuscript. The Central Hospital of Wuhan is a major tertiary hospital and is responsible for the treatment of COVID-19 patients. A total of 584 patients with confirmed COVID-19 in the Central Hospital of Wuhan from January 1 to February 15, 2020 were enrolled and diagnosed according to the interim guidance from the World Health Organization^18^. Respiratory tract samples including nasopharyngeal swabs and sputum were collected. Only cases confirmed by nucleic acid-positive for SARS-CoV-2 were included in this study. Five patients with chronic liver diseases, including chronic hepatitis B infection and cirrhosis, were excluded from this study. Twelve patients were excluded due to incomplete liver enzyme data on admission. Ultimately, 567 patients were included and separated into two groups according to their AST/ALT ratio on admission (< 1.38 or ≥ 1.38). Death was regarded as poor prognosis in this study. Of 567 patients, the survival data of twelve patients who were transfer to other hospital due to deterioration after clinical evaluation (at least PaO_2_/FiO_2_ < 300 mmHg and tracheal intubation) was unknown. Therefore, only 555 patients with clear endpoints (death or recovery) were enrolled in some analysis about prognosis.

### Procedures

The symptoms, signs, coexisting conditions, laboratory results, computed tomography (CT) findings, and outcomes were obtained from the medical records of enrolled patients in the Central Hospital of Wuhan. All patient testing was carried out according to clinical needs. The duration from onset of illness to hospital admission was recorded. The chest CT findings, laboratory data, Sequential Organ Failure Assessment (SOFA) scores, CURB-65 scores and Acute Physiology and Chronic Health Evaluation II (APACHE II) scores were measured on admission unless stated otherwise. Laboratory assessments consisted of a complete blood count, blood chemical analysis, liver and renal function evaluation, coagulation testing, and levels of C-reactive protein (CRP), procalcitonin, lactate dehydrogenase (LDH), creatine kinase (CK), creatine kinase-MB (CK-MB), lactate, and interleukin-6 (IL-6). Medical records were comprehensively reviewed and extracted by a standardized chart review form. The clinical outcomes were documented up to the final follow-up date of March 25, 2020.

### Statistics

Receiver operating curve (ROC) was utilized to compare the effects of different clinical indexes in predicting prognosis and find out cut-off value. Kaplan–Meier survival analysis was employed to show the outcome of COVID-19 patients. We described the categorical variables as numbers with percentages and continuous variables as medians with interquartile range (IQR) values. Categorical variables and count data were compared using the Chi-square tests, and Continuous correction chi-squared tests was performed if relevant data were limited. Normally distributed data of continuous variables were compared using independent group t-tests; when the data were not normally distributed, Mann–Whitney U tests were performed. Furthermore, univariate and multivariate logistic regression analysis was performed after collinearity diagnostics. A variance inflation factor (VIF) greater than 5 was considered significant collinearity. Spearman’s rank correlation analysis was performed to select variables for further multivariate analysis. Paired Wilcoxon's tests were performed to compare the AST/ALT ratio on different hospitalization days. All data were statistically analyzed using SPSS software, version 25.0. In this study, a two-tailed P value less than 0.05 was considered statistically significant.

### Ethics approval

This study was approved by the Ethics Committee of The Central Hospital of Wuhan.

## Abbreviations

ACE2: Angiotensin-converting enzyme 2
ALT: Alanine aminotransferase
APCHE II: Acute physiology and chronic health evaluation II
AST: Aspartate aminotransferase
BUN: Blood urea nitrogen
CK: Creatine Kinase
COVID-19: Coronavirus disease 2019
CRP: C-reactive protein
IL6: Interleukin-6
IQR: Interquartile Range
LDH: Lactate dehydrogenase
CT: Computed tomography
MERS-CoV: Middle east respiratory syndrome Coronavirus
SARS-CoV-2: Severe acute respiratory syndrome-Coronavirus 2
SOFA: Sequential organ failure assessment
VIF: Variance inflation factor
